# Drug Abstinence Self-Efficacy Scale (DASES): psychometric properties of the Farsi version

**DOI:** 10.1186/s13011-020-00336-9

**Published:** 2021-01-03

**Authors:** Maryam Khazaee-Pool, Seyed Abolhassan Naghibi, Tahereh Pashaei, Mosharafeh Chaleshgar-Kordasiabi, Mahbobeh Daneshnia, Koen Ponnet

**Affiliations:** 1 Department of Public Health, School of Health, Mazandaran University of Medical Sciences, Sari, Iran; 2 Health Sciences Research Center, Addiction Research Institutes, Mazandaran University of Medical Sciences, Sari, Iran; 3 Department of Public Health, School of Health, Kurdistan University of Medical Sciences, Sanandaj, Iran; 4 Department of Nursing and Midwifery Ramsar, School of Medicine, Babol University of Medical Sceinces, Ramsar, Iran; 5 Department of Communication Studies, imec-mict, Ghent University, Ghent, Belgium

**Keywords:** Drug abstinence self-efficacy scale, Psychometric, Reliability, Validity

## Abstract

**Background:**

Research has demonstrated that therapeutic interventions based on the self-efficacy theory produce positive outcomes for people who exhibit addictive behaviors, such as alcohol and drug use. Several questionnaires based on self-efficacy theory have been developed to evaluate the extent to which intervention programs can modify behavior. The present study describes the psychometric properties of the Farsi version of the Drug Abstinence Self-Efficacy Scale (DASES).

**Design and methods:**

The forward–backward approach was employed to translate the DASES from English into Farsi. A cross-sectional study was conducted, and the psychometric properties of the Farsi version of the DASES were measured. Using a cluster sampling method, 400 male people who use drugs aged 20 years or older were selected from 10 addiction treatment clinics in Mazandaran, Iran. The internal consistency and test–retest methods were used to measure the reliability of the DASES. Face and content validity were measured, and the construct validity of the DASES was assessed through both exploratory factor analysis (EFA) and confirmatory factor analysis (CFA). The data were analyzed using SPSS and AMOS.

**Results:**

The results of the EFA indicated a four-factor solution for the DASES that accounted for 64.72% of the observed variance. The results obtained from the CFA demonstrated that the data fitted the model: the relative chi square (× 2/df) equaled 1.99 (*p* < 0.001), and the root mean square error of approximation equaled 0.071 (90% CI = 0.059–0.082). All the comparative indices of the model were equal to or greater than 0.90 (0.91, 0.93, 0.94, 0.93, and 0.90, respectively). The Cronbach’s alpha ranged from 0.90 to 0.93, proving a satisfactory reliability. Additionally, the intraclass correlation coefficient ranged from 0.75 to 0.98, which is an acceptable result.

**Conclusions:**

This study’s results show that the Iranian version of the DASES has good psychometric properties and is appropriate for assessing substance use behaviors among Iranian addicted persons.

## Background

Substance use disorder, a chronic, persistent disease associated with personal, familial, and social dysfunctions, is one of the world’s more challenging health care problems [1–3]. The prevalence of substance use has been fixed at around 0.5% of the global population over the past decade, but it differs by area and type of drug used. East and Southeast Asia are among the main centers for the production and transportation of illegal drugs [4]. Iran shares a border with Afghanistan, a major manufacturer of narcotics, which facilitates the importation of illicit drugs to Iran [3, 5]. As in other Islamic countries, substance use is forbidden in Iran based on Islamic law, customs, and social values [6]. Despite this, Iran ranks high in opium use; about 1.8–3.3 million persons use drugs annually [3], and drug and alcohol disorders make up about 2% of the total disease burden [7]. As Iran is recognized as having among the highest rates of drug use for heroin, cannabis, and methamphetamine, it is not surprising that drinking alcohol and using drugs comprise almost 2% of the country’s disease burden [8].

Substance use prevention, particularly primary prevention, is the most economical and potentially effective solution to this problem [9], but clear indications of the characteristics of substance use disorder are a prerequisite for substance use prevention. Successful methods of ending drug use are often based on behavioral change models and theories, and two cognitive-behavioral theories have been suggested that may explain the relapse process and that offer important suggestions for designing effective management approaches [10, 11]. Albert Bandura developed the self-efficacy theory based on social cognitive theory, according to which individuals can influence their setting and environment rather than merely reacting to them. Self-efficacy aligns with this as it relies on individuals’ belief in their capacity to execute the behaviors needed to produce specific results [12]. According to the theory, if individuals do not believe they have the capacity to implement the behavior needed to reach the desired goal, they will put forth minimal effort or not engage in that behavior. Self-efficacy beliefs are also believed to vary depending on the domain of functioning and the circumstances of the behavior’s occurrence [10, 12].

The theory suggests that self-efficacy can alter behavior through the recognition of background signs and through encouragement to achieve a specific result. Effective interventions to decrease drug use or other addictive behaviors are supposed to strengthen efficacy beliefs related to a person’s ability to reduce the desire to take part in such behaviors [12]. Self-efficacy is a key factor in treating substance use as individuals must be confident of their ability to stop using drugs. Without self-efficacy, treatment can be challenging. In fact, people who use drugs may believe that they cannot stop using substances.

In the context of substance use, self-efficacy can influence a people’s ability to withdraw from drug use when they are close to other persons who consume drugs, are pressured by others to use, or are in specific settings [13]. It is often hard for people who use drugs to resist the temptation to use, and strong self-efficacy beliefs may help them resist [14].

Based on social cognitive theory, many instruments have been developed to assess the degree to which interventions can change personal behavior. One such scale in substance abstinence, based on the self-efficacy structure of Bandura’s social cognitive theory, is the Drug Abstinence Self-Efficacy Scale (DASES) [15]. DASES is a 20-item self-report survey that measures confidence in one’s ability to abstain from using drugs in specific situations. The test results can help in assessing whether an individual is ready for drug treatment and in determining the right mode of treatment. High scores on the DASES indicate that individuals have more confidence in their ability to abstain from drugs. Conversely, low scores indicate that they do not believe they can resist the temptation to use drugs [15]. This information can help medical professionals determine the treatment path and aid therapists in improving self-efficacy. DiClemente et al. (1994) developed the Alcohol Abstinence Self-Efficacy Scale (AASES), which was adapted by Hiller and colleagues (2000) [15] into the DASES [16]. The DASES has been validated in other countries [15], but no study has validated it in Iran.

As cultural and linguistic factors may impact how respondents complete the original version of the DASES, it is important to reevaluate the validity and reliability of DASES in different cultures, such as that of Iran. Therefore, the present study measures the psychometric properties of the Iranian version of the DASES to support abstinence in Iranian drug-addicted persons.

## Methods

### Sample and data collection

To evaluate the psychometric properties of the DASES among drug-addicted people aged 20 years and older, a cross-sectional study was conducted in Mazandaran, Iran, in November–December 2019. Twelve addiction treatment clinics in Mazandaran participated in the study. A cluster sampling method was used where each cluster contained the same number of respondents. First, Mazandaran was divided into three regions (east, west, and central), and all the addiction treatment clinics in them were identified. Next, four clinics were randomly selected from each region. Patients who were referred to the clinics were asked whether they were willing to participate in the study. Only patients over 20 years’ old who could read and write in Farsi were eligible to participate.

After the first author conducted a short interview and provided information on the study’s process, the patients who agreed to participate completed the DASES. They had been assured that their responses were anonymous and confidential and that they could terminate their participation at any time, whereupon they provided written consent. The participants’ demographic characteristics, including age, employment status, educational level, marital status, and age when starting drug use, were also collected (see Table 1). The participants answered a paper-and-pencil questionnaire, which took 20–25 min to complete.

**Table 1:** Characteristics of the study sample

	**EFA sample** **(***n*** = 200)**	**CFA sample** **(***n*** = 200)**	**Test–retest** **sample (***n*** = 35)**
	**Number (%)**	**Number (%)**	**Number (%)**
**Age (years)**			
20–29	39 (19.5)	47 (23.5)	16 (45.7)
30–39	86 (43)	77 (38.5)	9 (25.7)
≥ 40	75 (37.5)	76 (38)	10 (28.6)
**Employment status**			
Unemployed	68 (34)	61 (30.5)	19 (54.3)
Part time job	72 (36)	84 (42)	7 (20)
Full time job	60 (30)	55 (27.5)	9 (25.7)
**Educational Level**			
Primary	47 (23.5)	43 (21.5)	9 (25.7)
Secondary	134 (67)	129 (64.5)	14 (40)
Higher	19 (9.5)	28 (14)	12 (34.3)
**Marital status**			
Single	64 (32)	68 (34)	10 (28.6)
Married	81 (40.5)	95 (47.5)	14 (48.5)
Divorced / widowed	55 (27.5)	37 (18.5)	8 (22.9)

Basic sample demographics broken down by EFA, CFA, and Test–retest samples

## Measurement

### The drug abstinence self-efficacy scale (DASES)

The DASES [15] is a modified version of the AASES, which was devised in 1994 by DiClemente et al. [16]. Due to the relative lack of tools to assess self-efficacy in drug abstinence, Hiller et al. (2000) tested the AASES questionnaire on a sample of people who use drugs and estimated its psychometric properties. The DASES assesses an individual’s efficacy (e.g., confidence) in abstaining from drugs in 20 typical drug-taking situations. It has been confirmed as an effective self-measurement scale that can lead to improved motivation for changing behavior [17, 18]. Individuals are asked to estimate their current efficacy in abstaining from drugs. The situations embrace four subscales and are rated on a 5-point Likert scale ranging from *not at all* (0) to *extremely* (4), with the total score ranging from 0 to 80 and higher scores indicating greater self-efficacy in abstaining from drugs [15]. These scales may also be used to evaluate personal treatment, the progress of drug treatment, relapse potential, and post-treatment functioning. Hiller et al. found a Cronbach’s alpha of 0.90 for the 20-item DASES and Cronbach’s alphas of 0.92, 0.92, 0.89, and 0.87 for the subscales (Negative Affect, Social Pressure, Cravings and Urges, and Physical and Other Concerns about Using Drugs, respectively) [15].

### Translation procedures

The authors received permission from the developers of DASES to translate it from English into Farsi by applying a forward–backward approach [19]. First, two independent, professional, native Farsi translators translated the DASES into Farsi. Next, those two versions were compared by one of the authors and both the translators and aggregated into a single version of the DASES. In the backward phase, two other persons who spoke fluent Farsi and English translated the Farsi version of the DASES back into English, and a provisional English version of the DASES was made. The accuracy of the back-translated DASES was then evaluated. To measure the content validity, a panel of experts (an epidemiologist, a health promotion expert, and an expert in drug control) compared the provisional English version of the DASES with the original instrument. The final Iranian version of the DASES was then produced [20].

### Psychometric testing

To examine the psychometric properties of the Iranian version of the DASES, we assessed its content, face, and construct validity as well as its reliability and stability.

### Content validity

Both qualitative and quantitative approaches were used to assess the content validity. In the qualitative phase, a panel of experts, including health promotion experts, epidemiologists, and specialists in drug-use control, measured the content validity of the DASES, evaluating its phrasing, grammar, wording, item allocation, and scaling. In the quantitative phase, the content validity index (CVI) and content validity ratio (CVR) of the DASES were assessed. The CVI was assessed by asking the experts to rate each item according to its simplicity, relevance, and clarity [21] on a scale from 1 = *not relevant, simple, or clear* to 4 = *very relevant, simple, and clear*. The CVI was measured as the proportion of items on the questionnaire that achieved a rating of 3 or 4 [22, 23]. The essentiality of each item in the questionnaire was evaluated by the CVR. To measure the CVR, the specialists rated each item as 1 = *essential*, 2 = *useful but not essential*, or 3 = *not essential*. Afterward, according to the Lawshe table, items with a CVR score of 0.62 or greater were determined to be acceptable and were maintained [23].

### Face validity

Both a qualitative and a quantitative approach were used to measure face validity. A sample of addicted males (*n* = 10) was asked to assess each item of the Iranian version of the DASES, and indicate whether they experienced difficulty or confusion in answering it. Afterward, the impact score (importance × frequency) was measured to determine the percentage of addicted persons who recognized an item as important or quite important on a 5-point Likert instrument. An item was determined to be suitable if it had an impact score of 1.5 or higher (which equals a mean rate of 50% and a mean importance of 3 out of 5) [24].

### Construct validity

#### Exploratory factor analysis

An EFA was performed to find the main factors of the DASES. The sample size was assessed a priori and was estimated according to the number of items in the instrument multiplied by 10 (10 × 20 = 200) [25]. The participants were recruited from the 12 clinics. In a section below, we describe the characteristics of the sample. A principal component analysis with varimax rotation was applied to extract the main factors. Factor loadings of ≥ 0.40 were considered acceptable [26].

#### Confirmatory factor analysis

A CFA was performed to measure the coherence between the model and the data. Considering the possible attrition related to test–retest analysis, we planned to recruit a separate sample of addicted persons from the 12 clinics in Mazandaran. Assigning 10 individuals to each item, a sample size of 200 was estimated [27]. To assess the model fit, the relative chi square, comparative fit index (CFI), goodness of fit index (GFI), root mean square error of approximation (RMSEA), standardized root mean square residual (SRMR), normed fit index (NFI), and non-normed fit index (NNFI) were measured [28, 29]. CFI, GFI, NNFI, and NFI values range from 0 to 1, but values equal to or greater than 0.80 are generally considered satisfactory model fits. An RMSEA value between 0.08 and 0.10 indicates a mediocre fit, and a value lower than 0.08 indicates a good fit. Values of less than 0.05 indicate a good fit for the SRMR while values between 0.05 and 0.08 and between 0.08 and 0.10 represent a close fit and a satisfactory fit, respectively [30].

### Internal consistency and homogeneity

The internal consistency was assessed using the Cronbach’s alpha coefficient to measure the reliability of the DASES. Alpha values equal to or greater than 0.70 were considered acceptable [31]. Moreover, the item-total correlations and mean inter-item correlations were contained within the analysis. Westen (2005) advises that it is necessarily to assess the inter-item correlation as a criterion for internal consistency and suggests that all-person inter-item correlations should be 0.15 or greater [32]. One-dimensionality can be confirmed only if the all-person inter-item correlations are grouped approximately near the mean inter-item correlation. The corrected item-total correlation is the correlation of the item chosen with the accumulated score for all the other items [33]. Thus, the corrected item-total correlation was applied.

### Stability

Additionally, the intraclass correlation coefficient (ICC) was assessed to evaluate the stability of the DASES, which was re-administered to 30 addicted men two weeks after the first completion. ICC values equal to or greater than 0.40 are considered satisfactory (*r* values between 0.81 and 1.0 are excellent, those between 0.61 and 0.80 are very good, those between 0.41 and 0.60 are good, those between 0.21 and 0.40 are fair, and those between 0.0 and 0.20 are poor) [31]. The analyses were performed using the statistical program SPSS for Windows version 24.0 and Amos 24.0.

## Results

### Demographic characteristics of the participants

In this study, 400 addicted men aged 20 years or older completed the DASES (200 participants for EFA and 200 for CFA). The largest group in the sample comprised men aged 30–39 years (40.75%). Regarding employment status, 32.25% (129 participants) were unemployed, 39% (156 participants) had part-time jobs, and 28.75% (115 participants) worked full time. Most of the participants (65.75%) had a secondary level of education, and 44% of the sample were married. Table 1 provides an overview of the descriptive characteristics of sample 1 (EFA), sample 2 (CFA), and sample 3 (test–retest).

### Content validity

The translated DASES was arbitrated for relevance and for the phrasing of the items by the expert panel members, who could suggest practical phrasing enhancements for each item. Subsequent to this, the revision of the Farsi DASES was prepared and debated once more by the expert panel until agreement was reached on the content.

### Construct validity

#### Exploratory factor analysis

**Fig. 1:**
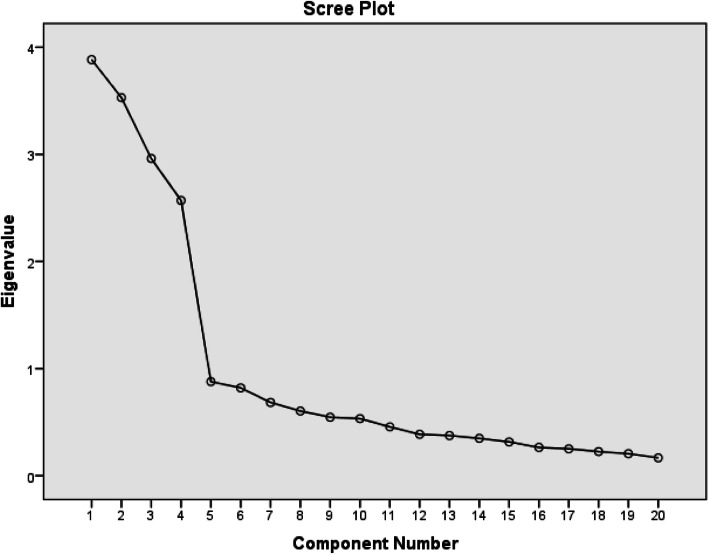
Scree plot for determining the factors. Scree plot from the principal components analysis in the EFA sample

An EFA was conducted on the 20 items of the DASES (cutoff point: 0.50). The assessed Kaiser–Meyer–Olkin coefficient was 0.792, with a p value of < 0.001, demonstrating that the sample was large enough to provide a satisfactory principal component analysis with varimax rotation. In the 20-item scale, four factors revealed eigenvalues greater than 1, explaining 64.72% of the variance, and the scree plot also revealed a four-factor solution (see Fig. 1).

The factor loadings of the 20 items were 0.40 or more, ranging from 0.59 to 0.88. The factor loadings of each item and the four dimensions are presented in Table 2. All the items were loaded on their own dimensions.

**Table 2:** Exploratory factory analysis of the DASES (*n* = 200)

**Item**				
**Negative Affect**				
Q6: When I am very worried	**.844**	.045	.070	.006
Q3: When I am feeling depressed	**.844**	.099	.004	-.101
Q14: When I feel like blowing up because of frustration	**.836**	.012	.096	.041
Q16: When I sense everything is going wrong for me	**.834**	-.012	.011	.036
Q18: When I am feeling angry inside	**.788**	-.037	.012	-.070
**Social Pressure**				
Q15: When I see others using drugs at a bar or a party	.005	**.882**	-.013	.045
Q4: When I am on vacation and want to relax	-.002	**.876**	.101	.145
Q8: When I am offered a drug in a social situation	-.007	**.832**	-.016	.107
Q17: When people I used to drink with encourage me to use drugs	-.027	**.774**	.119	.099
Q20: When I am excited or celebrating with others	.106	**.628**	-.019	-.067
**Physical and Other Concerns about Using Drugs**				
Q2: When I have a headache	.004	-.029	**.865**	.032
Q13: When I am experiencing some physical pain or injury	-.016	.060	**.829**	-.029
Q5: When I am concerned about someone	.089	.157	**.782**	.006
Q9: When I dream about using a drug	.048	.060	**.781**	.034
Q12: When I am physically tired	.056	-.077	**.710**	-.009
**Cravings and Urges**				
Q10: When I want to test my willpower over using drugs	.034	.091	-.027	**.834**
Q7: When I have the urge to try just one drug use to see what happens	.002	.010	.015	**.812**
Q11: When I am feeling a physical need or craving to use drugs	-.030	.094	.028	**.785**
Q1: When I am in agony because of stopping or withdrawing from drug use	-.003	.025	-.058	**.750**
Q19: When I experience an urge or impulse to take a drug that catches me unprepared	-.065	.050	.059	**.597**

Factor loadings for four extracted principal components in the EFA sample following a varimax rotation

#### Confirmatory factor analysis

**Fig. 2:**
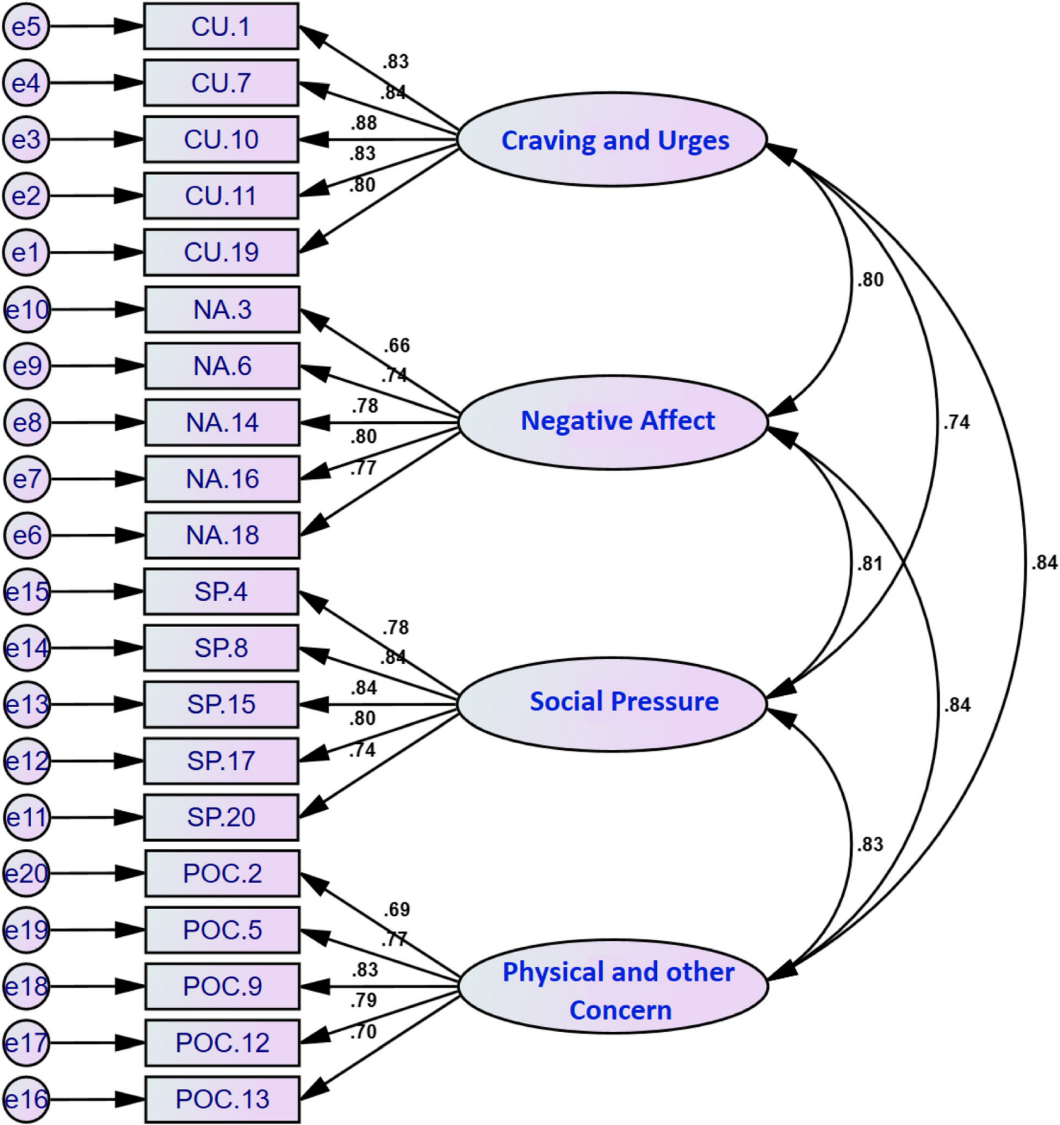
A four-factor model for the DASES obtained from CFA (*n* = 200). Schematic of the four-factor solution using the CFA showing loadings and inter-factor correlations

We conducted a CFA on the 20-item DASES to assess the fitness of the model obtained from the EFA, and covariance matrixes were analyzed. All the fit indices were found to be appropriate. The relative chi square (χ2/df) equaled 1.99 (*p* < 0.001). The RMSEA of the model was 0.071 (90% CI = 0.059–0.082), and the SRMR was 0.071. All the comparative indices in the structural model, i.e., GFI, AGFI, CFI, NNFI, and NFI, were greater than 0.90 (0.91, 0.93, 0.94, 0.93, and 0.90, respectively). Even though the model fit was acceptable and good, the modification indices of the regression weights were analyzed to find the covariance among the four factors. The model could not be improved, however, so no variations were performed, and the model was accepted in its existing form. Figure 2 demonstrates the model.

## Reliability

### Internal consistency

The DASES completed by the 200 participants who use drugs were used in the analyses. The instrument had an overall Cronbach’s coefficient alpha of 0.96, and the inter-item correlations ranged from 0.61 to 0.78. Afterward, the alpha coefficient values for the items were measured, which indicated that the internal consistency level of the DASES was 0.96 and ranged from 0.81 for the Negative Affect and Physical and Other Concerns about Using Drugs sub-scales to 0.82 for the Social Pressure and Cravings and Urges sub-scales, which is greater than the acceptable threshold (Table 3).

**Table 3:** Item-total correlations of items of the scale (n: 200)

	Scale Mean if Item Deleted	Scale Variance if Item Deleted	Corrected Item-Total Correlation	Cronbach's Alpha if Item Deleted
Q.1	76.67	106.494	.747	.954
Q.2	76.68	110.249	.617	.956
Q.3	76.70	109.928	.618	.956
Q.4	76.57	108.580	.704	.955
Q.5	76.69	108.130	.718	.955
Q.6	76.57	108.569	.671	.955
Q.7	76.58	107.099	.734	.954
Q.8	76.60	107.107	.729	.954
Q.9	76.64	108.002	.743	.954
Q.10	76.61	105.521	.783	.954
Q.11	76.61	104.938	.730	.954
Q.12	76.68	106.332	.733	.954
Q.13	76.67	106.794	.671	.955
Q.14	76.53	108.063	.709	.955
Q.15	76.56	107.570	.723	.954
Q.16	76.56	107.935	.713	.955
Q.17	76.57	107.434	.720	.954
Q.18	76.57	107.371	.702	.955
Q.19	76.59	105.472	.731	.954
Q.20	76.53	107.323	.699	.955

Item performance metrics for each of the DASES items

## Stability

Additionally, a test–retest analysis was conducted to assess the stability of the DASES. The results found a satisfactory threshold. The ICC ranged from 0.75 to 0.98 for the dimensions of the DASES, supporting the scale’s stability. The results are presented in Table 3.

## Discussion

This study examined the factor validity, dimensionality, and reliability of the DASES among Iranian people who use drugs. Overall, the findings indicate that the psychometric characteristics of the Farsi version of the DASES are good. Consistent with other studies, we found a four-factor structure [15, 16], indicating that improvement in self-efficacy happens through several points and consequently demonstrating the role of individual dissimilarities, which urges further study within this population. Furthermore, the development of theory-based scales can serve as an important prerequisite for the assessment of any educational program. Consequently, we consider the results of the present study to be valuable for clients who are part of a drug-control plan.

Overall, the study found satisfactory psychometric properties for the DASES, with the CVI and CVR indicating that its content validity was good. Furthermore, the results of the EFA and CFA revealed a good structure for the DASES, with the EFA showing that the four-factor structure of the DASES accounted for 64.72% of the total observed variance. The results of the EFA were compatible with those achieved by the English version of the DASES. This shows that the DASES is useful for revealing various aspects of the health concerns influenced by drug use. As expected, this study indicated a four-factor solution for the Iranian version of the DASES, including Negative Affect, Social Pressure, Cravings and Urges, and Physical and Other Concerns about Using Drugs subscales.

The CFA also examined whether coherence exists between the information and the theoretical structure. The CFA revealed good fit indices for the existing model and demonstrated the acceptable convergent validity of the four subscales of the DASES (i.e., Negative Affect, Social Pressure, Cravings and Urges, and Physical and Other Concerns about Using Drugs). These findings related to the CFA are consistent with the model from the original instrument developed by Hiller et al. (2000) [15], showing that the DASES is reliable when used by Farsi-speaking addicted persons.

Additionally, acceptable levels of the Cronbach’s alpha and the ICC were found, and the good stability and reliability of the DASES were demonstrated. The internal consistency of the DASES, as evaluated by the Cronbach’s alpha, displayed a suitable reliability for the four dimensions in accordance with the original study [15]. Moreover, after 35 male participants who use drugs were tested over a two-week period, the test–retest reliability coefficient of the DASES was a satisfactory 0.78. It is generally held that assessments of repeatability for group comparisons should be at least 0.70 [17, 34], so our results clearly show that the DASES has appropriate stability in the short term; however, it has yet to be determined whether it is stable over the long term. Overall, the findings indicate satisfactory psychometric properties for the DASES.

The DASES provides an understanding of the processes by which addicted persons attempt to modify their addictive drug use behavior. The development of theory-based scales serves as an important prerequisite for the assessment of any educational program. Consequently, we consider the results from the present study to be valuable for clients who are taking part in a drug-control plan.

### What is already known on this topic

Existing studies reveal that the incidence of substance use is increasing worldwide [35], and increasing attention has been paid to the effect of self-efficacy as a predictor and/or intermediary of remedy results in numerous areas. In several studies of drug use remedies, self-efficacy has appeared as a significant predictor of the result or as an intermediary of the remedy’s influences [36]. Consequently, the DASES for this condition is crucial to prevention inventions [15].

### What this study adds

The Farsi version of the DASES may provide a valid scale for Iranian patients with substance use difficulties. It displays statistically satisfactory levels of validity and reliability.

## Limitations

Although the results of the current study reveal several benefits, some limitations must be considered. The first concerns the truthfulness of the clients’ responses due to the self-reported nature of the answers. The generalizability and sample size constitute other limitations. The sample was limited to a group of 400 (both EFA and CFA) men who use drugs, and it is uncertain whether we would attain the same results if a larger sample of both male and female participants who use drugs was employed. Consequently, the present findings may not be able to measure gender differences regarding the psychometric properties of the DASES. In future studies with a larger group of both male and female people who use drugs, researchers should consider measuring whether motivations to cease drug use are similar between the genders, whether gender affects acceptance of treatment, and whether the current findings remain valid. Furthermore, it would be interesting for future studies with a larger sample to test whether the psychometric properties of the instrument persist with alternative measures of reliability and validity (e.g., test–retest validity). Finally, the present study included only addicted persons who were referred to clinics. Future studies should also measure the psychometric properties of the Iranian version of the DASES in Iranian addicted persons who were referred to drop-in centers.

## Conclusion

The findings suggest that the Farsi version of the DASES is a valid, reliable measure to assess drug use among Iranian addicted men. The DASES is important because it provides standardized information about substance use self-efficacy behaviors. The use of a procedure and method accepted in the scientific literature makes available the assessment of information garnered from diverse backgrounds. It is suggested that the DASES should be further assessed in both dissimilar areas of Iran and in different populations and cultures. When a valid and reliable instrument is devised, it may be applied to assess the consequences of intervention research, and, as previously stated, it should be assessed among dissimilar populations and backgrounds. The scale that was assessed in the current study will contribute positively to the progress of more effectual, evidence-based anti–substance-use plans for the population. Furthermore, the Farsi version of the DASES may help health care workers to find and plan health strategies that are useful and targeted to patients of particular statuses.

## Data Availability

The datasets produced and analyzed in the present study are not publicly available in order to preserve the participants’ privacy, but they are available from the corresponding author upon reasonable request.

## Abbreviations

DASES: Drug abstinence self-efficacy scale
AASES: Alcohol Abstinence Self-Efficacy Scale
DIC: Drop in Center
CVI: Content validity index
CVR: Content validity ratio
EFA: Exploratory factor analysis
CFA: Confirmatory factor analysis
GFI: Goodness of Fit Index
CFI: Comparative Fit Index
RMSEA: Root Mean Square Error of Approximation
NNFI: Non-Normed Fit Index
NFI: Normed Fit Index
SRMR: Standardized Root Mean Square Residual
ICC: Intraclass correlation coefficient
